# Six Revision Surgeries for Massive Epidural Fibrosis with Recurrent Pain and Weakness in the Left Lower Extremity

**DOI:** 10.3390/medicina58030371

**Published:** 2022-03-02

**Authors:** Ho Yong Choi, Dae Jean Jo

**Affiliations:** Department of Neurosurgery, Kyung Hee University Hospital at Gangdong, Kyung Hee University of Medicine, Seoul 05278, Korea; apuzzo@hanmail.net

**Keywords:** epidural fibrosis, discectomy, failed back surgery syndrome, intervertebral disc

## Abstract

Epidural fibrosis is a common cause of pain after lumbar surgeries. There are no previous reports documenting profound limb weakness associated with epidural fibrosis. A 43-year-old woman uneventfully underwent microscopic discectomy. However, six additional surgeries were needed due to recurrent pain and weakness episodes, several days after the surgery. Operative findings were severe epidural fibrosis around the thecal sac and nerve roots. Epidural fibrosis excision did not prevent recurrent fibrosis; therefore, we performed a lordotic fusion with posterior column shortening to reduce neural tension and nerve-root stretching. Eventually, she became free from recurrent episodes of deteriorations and repetitive surgeries.

## 1. Introduction

Lumbar discectomies are surgical procedures that are commonly performed in patients with herniated lumbar intervertebral discs. Previous studies have reported that 20–40% of patients have persistent or recurrent sciatica or intractable back pain [1,2,3,4,5,6,7,8,9]. Epidural fibrosis is known to be a common cause of pain following lumbar surgeries and is also associated with failed back surgery syndrome (FBSS) [10,11,12,13,14]. Here, we reported on a patient suffering from recurrent episodes of pain and neurological deterioration associated with massive epidural fibrosis following lumbar discectomy. Five subsequent revision surgeries for epidural fibrosis removal could not cease the worsening of recurrent episodes. Thus, we performed a lordotic fusion with posterior column shortening to reduce neural tension and nerve root stretching. Informed consent was obtained from the patient for the publication of this case.

## 2. Case Presentation

**Figure 1:**
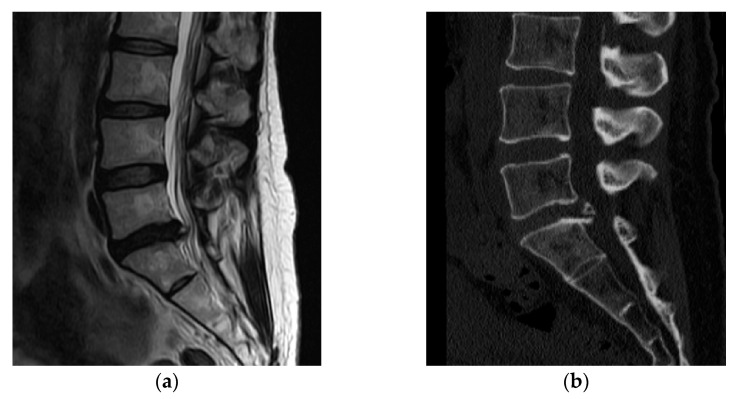
A 43-year-old woman presented with a radiating pain in her left lower extremity. (**a**) Magnetic resonance imaging (MRI) showed left central herniated disc at the L5/S1 level. (**b**) Computed tomography (CT) revealed a combined posterior ring apophysis fracture at the same level.

A 43-year-old woman presented to our hospital with a radiating pain in her left lower extremity. The visual analogue scale (VAS) score in her legs was 9. Magnetic resonance imaging (MRI) showed an L5/S1-level left central herniated disc. Computed tomography (CT) revealed combined posterior ring apophysis fracture at the same level (Figure 1). Conservative management did not work; therefore, she underwent microscopic discectomy. Postoperatively, she was discharged uneventfully, with improvements in the radiating pain.

**Figure 2:**
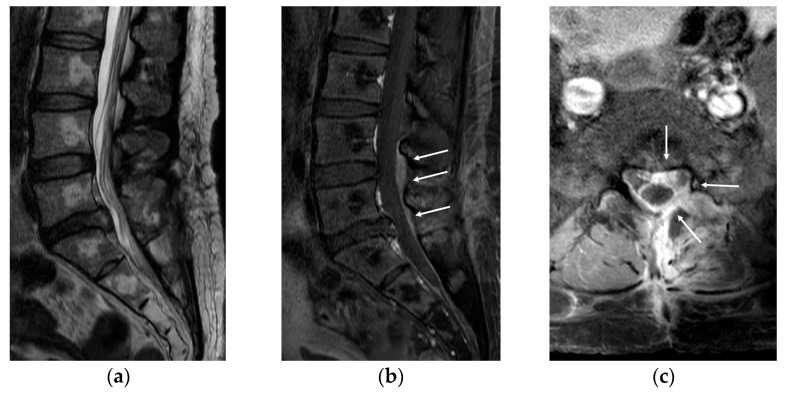
Magnetic resonance imaging (MRI) was conducted to assess the recurrent pain in her back and left lower extremity at 11 days after surgery. (**a**) MRI revealed no evidence of recurred or remnant disc. (**b**,**c**) However, diffuse enhancement was noted at the posterior epidural space from L4 to S1 levels (arrows) and around the peridural space at the L5/S1 level (arrows).

Eleven days after surgery, she was admitted to the emergency room due to severe pain in her back and left lower extremity. There was no clinical evidence of systemic infection (normal body temperature and no elevation of inflammatory marker) or wound problem (clear wound without local heating, redness, or discharge). MRI revealed no evidence of recurred or remnant disc. However, diffuse enhancement at the posterior epidural space from L4 to S1 levels and around the peridural space at the L5/S1 level (Figure 2) was noted. Conservative treatment, including root block, did not work; therefore, we decided to conduct revision surgery (L5/S1 posterior lumbar interbody fusion).

**Figure 3:**
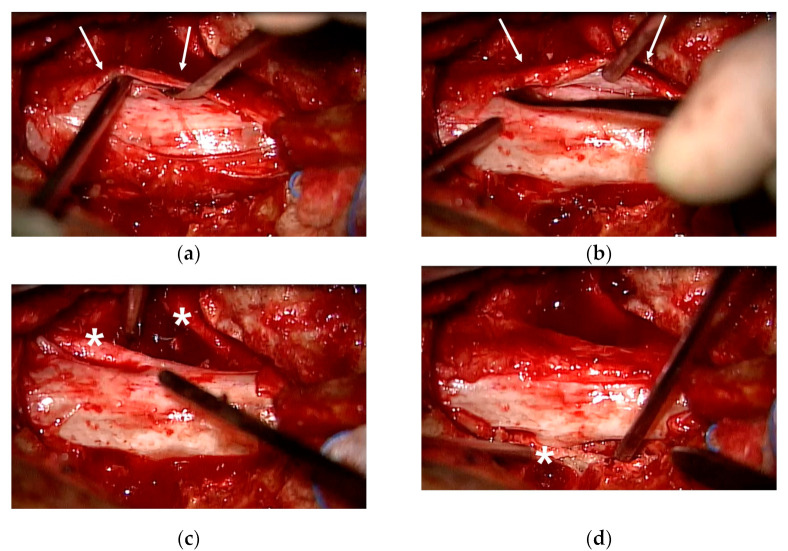
(**a**,**b**) Revision surgery revealed severe epidural fibrosis along the thecal sac and L5–S1 roots. Normal dura was identified following thick epidural fibrosis peeling (arrows). (**c**,**d**) Bilateral lumbosacral roots (asterisks) were identified and released by fibrotic membrane removal.

**Figure 4:**
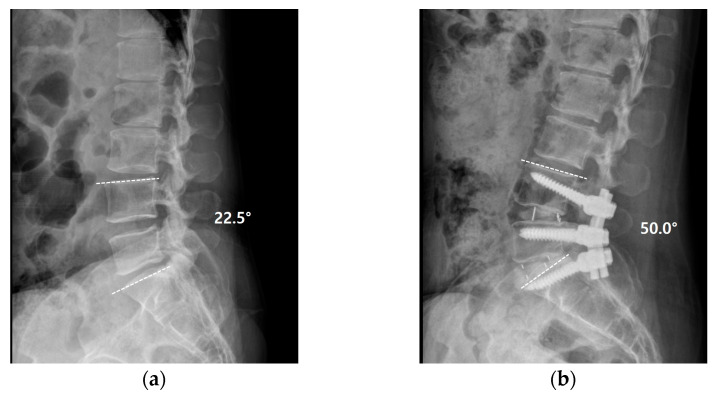
(**a**,**b**) Lordotic fusion from L4 to S1 with posterior column shortening was conducted to reduce neural tension and stretching of the nerve roots. Lower lumbar lordosis (L4–S1) was increased from 22.5° to 50.0° after surgery.

**Figure 5:**
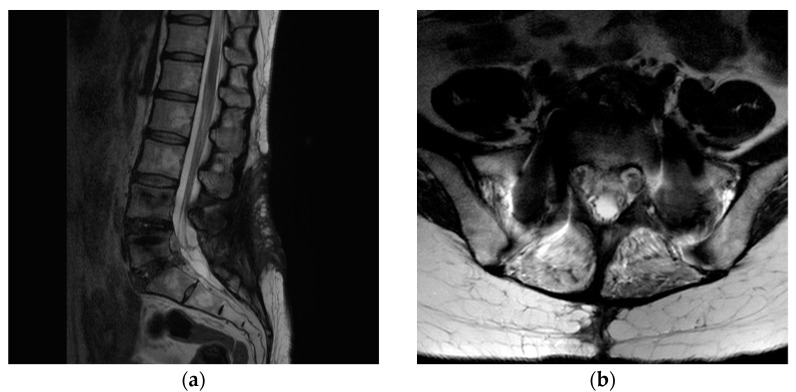
(**a**,**b**) Magnetic resonance imaging at 6 months postoperatively showed a well-decompressed thecal sac and nerve roots. However, roots were clumped and distorted within the thecal sac, suggesting arachnoiditis.

Five days after surgery, she complained of severe pain in her left lower extremity again. Following wound exploration with additional laminectomy, revealing no significant compressive lesion except epidural fibrosis, the symptom improved. Six days after revision surgery, she complained of gradually increasing pain in her left lower extremity, followed by profound weakness (no voluntary movement in the left ankle and big toe dorsiflexion and ankle plantarflexion). Revision surgery revealed severe epidural fibrosis along the thecal sac and L5–S1 roots (left side dominant). Normal dura was identified after peeling the thick epidural fibrosis (Figure 3). Pain and weakness of the left extremity disappeared immediately after surgery. However, two additional surgeries were needed due to the same pain and weakness episodes, several days following the surgeries. Operative findings were also similar to that of the previous surgeries with severe epidural fibrosis around the thecal sac and nerve roots. Symptoms were also alleviated immediately after surgery. Postoperative steroid therapy or narcotics did not work at all. Laboratory and culture studies documented no evidence of infection or rheumatoid disease. Finally, we performed L4–S1 oblique lateral interbody fusion (OLIF) with hyperlordotic cages to ameliorate tethering and traction of the thecal sac and nerve roots through posterior column shortening (Figure 4). Pain and paresthesia were considerably decreased. Weakness was also reduced. Eventually, the patient was able to walk again following six additional surgeries over 11 weeks. After the final surgery, postoperative wound infection by *Candida albicans* was identified, and treated successfully with antifungal agents. Follow-up MRI at 6 months postoperatively showed a well-decompressed thecal sac and nerve roots. However, roots were clumped and distorted within the thecal sac, suggesting arachnoiditis (Figure 5). Fortunately, her pain in the leg was considerably reduced, without neurological deficits. At the final follow-up, she was doing well with oral medication without taking narcotics (VAS 3) over the 2 years postoperatively.

## 3. Discussion

We have presented a case of multiple revision surgeries following discectomy, resulting from recurrent episodes of postoperative pain and weakness in the left lower extremity. Although surgical treatment should be considered as the last resort in a case of FBSS [15,16,17], revision surgeries were inevitable here due to the profound weakness in the ankle and big toe (no voluntary movement at all). No evidence of postoperative hematoma or rheumatoid disorder during repetitive surgeries was documented. Except for postoperative fungal infection after the last operation, operative findings and laboratory studies identified no evidence of wound infection. To the best of our knowledge, the present study is the first documented report of repetitive spinal surgeries without identifiable postoperative complications.

During the repetitive surgeries, the only intraoperative abnormal findings were exaggerated tense dura mater with thick fibrosis and fixed nerve roots. Therefore, postoperative epidural fibrosis was thought to have caused the recurrent episodes of pain and neurological deficit. Previous studies have reported that efforts of repeat decompression and neurolysis are discouraging [15,16,17]; thus, the excision of epidural fibrosis did not prevent recurrent fibrosis in this study. Therefore, we decided to preform OLIF with hyperlordotic cage, shortening the posterior elements by increased lordosis. After lordotic fusion, the patient finally became free from repetitive surgeries.

Epidural fibrosis is known to be a common cause of pain following lumbar spinal surgery, and has been implicated in 8% to over 60% of cases of post-laminectomy syndrome [10,11,12,13,14]. However, the role of epidural fibrosis in pain generation has not fully been established. Some researchers have reported that most patients do not develop symptomatic complaints from epidural fibrosis, and a minority of patients have developed significant pain due to epidural fibrosis [15,17]. Annertz et al. reported that there were no differences regarding the presence and extent of epidural fibrosis in MRI between symptomatic and asymptomatic patients [15]. In this study, we concluded that epidural fibrosis resulted in recurrent symptoms because of the presence of thick epidural adhesion surrounding and compressing normal dura intraoperatively and the immediate relief of symptoms after revision surgeries.

In epidural fibrosis, fibrous scar tissue replaces the epidural fat and can cause compression of the dura mater and nerve roots, leading to their adherence and tethering to the surrounding structures [18]. In such conditions, repetitive mechanical force from stretching/slackening and pendulum movements could concentrate on the nerve roots, leading to nerve irritation [19].

Blood supply to the nerve roots could be compromised by fibrotic change and dura mater compression. In contrast to the blood supply of the peripheral nerves, which is mostly transported by intrinsic vasculature, more than half of the nerve roots are transported from the extrinsic system via diffusion through the cerebrospinal fluid [20]. In the case of epidural fibrosis, therefore, perineural microcirculation could be significantly compromised due to increased neural tension and decreased extrinsic blood supply. Stretching of the sticking roots could also decrease intraradicular blood flow. In the presence of periradicular adhesive tissue, nerve root movement is limited, and blood flow could be reduced to more than 70% during the straight leg raising (SLR) test [21,22]. In this study, the patient presented severe limitation in the SLR test, with clinical deteriorations.

Recurrent episodes of profound weakness in left lower extremity, in the setting of epidural fibrosis, are quite strange. We could not find any previous reports with epidural-fibrosis-associated limb weakness. However, one report exists demonstrating a decreased blood flow to the nerve roots being associated with electrophysiological deterioration during the SLR test. Takamori et al. reported that the amplitude of compound muscle action potential (CMAP) deteriorated significantly after the nerve stretching test. Significant correlations were found between the deterioration ratio of nerve-root blood flow and CMAP [23]. The decreased CMAP amplitude caused by sticking-root stretching could explain the lower extremity weakness, although an electrophysiological study was not performed in the present case.

The possible mechanisms of repetitive episodes of the patient’s clinical deterioration were supposed to be a combination of mechanical stress and impaired blood supply resulting from epidural fibrosis, especially with markedly increased susceptibility stresses due to unknown reasons. Repetitive excision of epidural fibrosis could not cease the vicious cycle; therefore, we performed lordotic fusion to reduce neural tension and stretching of the nerve roots. Eventually, the patient became free from recurrent episodes of clinical deteriorations and repetitive surgeries. Therefore, in a case of suspicious epidural fibrosis combined with recurrent episodes of clinical deteriorations, lordotic fusion with posterior column shortening may be a treatment option.

## 4. Conclusions

In the present study, we reported a patient suffering from recurrent episodes of clinical deterioration with massive epidural fibrosis following lumbar surgeries. The possible mechanisms were a combination of mechanical stress and impaired blood supply resulting from epidural fibrosis, with markedly increased susceptibility for stressed patients. In a case of suspicious epidural fibrosis combined with recurrent episodes of clinical deteriorations, lordotic fusion with posterior column shortening may be a treatment option.

## Data Availability

Not applicable.
